# Green analytical quality by design RP-HPLC technique with fluorimetric detection for simultaneous assay of cinnarizine and dompridone

**DOI:** 10.1098/rsos.240829

**Published:** 2024-07-17

**Authors:** Neamat T. Barakat, Amina M. El-Brashy, Mona E. Fathy

**Affiliations:** ^1^ Department of Pharmaceutical Analytical Chemistry, Faculty of Pharmacy, Mansoura University, Mansoura 35516, Egypt

**Keywords:** cinnarizine, domperidone, full factorial design, high-performance liquid chromatography, greenness, co-formulated tablets

## Abstract

A green and sensitive factorial design-assisted reversed-phase high-performance liquid chromatography (RP-HPLC) approach with fluorimetric detection has been developed for simultaneous determination of cinnarizine and domperidone in pure materials, commercial single and combined tablets. Both drugs are co-formulated in one dosage form and are commonly used for the effective treatment of motion sickness in the ratio of 4 : 3 for cinnarizine and domperidone, respectively. To achieve the developed method’s best performance and reliability, a 2^3^ full factorial design was applied during the optimization of chromatographic conditions. Subsequently, isocratic elution on a C18 column with a mobile phase mixture of methanol and 0.02 M potassium dihydrogen phosphate buffer (85 : 15 v/v, pH 3.0) was performed to achieve the best separation. Moreover, the flow rate of 0.8 ml min^−1^ was applied during the analysis with fluorescence detection at λ_ex_ 283 nm/λ_em_ 324 nm. The elution process was time effective; it consumed less than 6 min for a complete run as retention time for cinnarizine and domperidone, was 4.5 and 3.5, respectively. Good linearity of the proposed method was achieved within the concentration ranges of 0.2–4.5 and 0.4–5.0 µg ml^−1^ for domperidone and cinnarizine, respectively. The suggested approach was carefully validated according to the International Council for Harmonization (ICH) guidelines. Moreover, green analytical procedure index (GAPI), national environmental methods index (NEMI), analytical greenness (AGREE) and analytical eco-scale methods were applied to assess and confirm method greenness.

## 1. Introduction

Over the past few decades, high-performance liquid chromatography (HPLC) replaced various spectroscopic techniques in testing the pre-sale process, drug marketing and drug control [1]. Moreover, application of the design of experiments (DoE) approach for optimization of chromatographic conditions guaranteed procedure best performance and produced data reliability [2].

**Figure 1.**
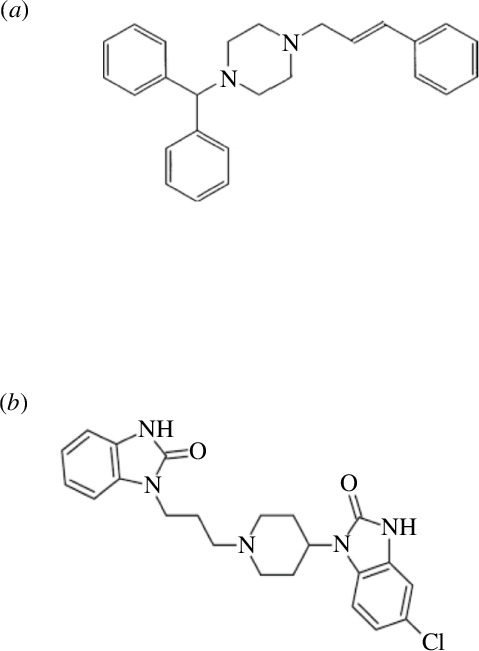
Chemical structure of (*a*) cinnarizine and (*b*) domperidone.

Cinnarizine (CRZ) is chemically defined; 1-benzhydryl-4-[(E)-3-phenylprop-2-enyl]piperazine [3] (figure 1*a*). It is a piperazine derivative with antihistamine and calcium-channel blocking activity. It is used for symptomatic treatment of nausea and vertigo caused by Meniere’s disease and for the prevention and treatment of motion sickness [4].

Domperidone (DPN) is chemically defined; 5-chloro-1-[1-[3-(2-oxobenzimidazolin−1-yl)propyl]-4-piperidyl]benzimidazolin-2-one, (figure 1*b*) [3]. It is a dopamine antagonist used as an antiemetic for short-term treatment of nausea and vomiting of various etiologies. It is neither considered suitable for chronic nausea and vomiting nor for the routine prophylaxis of postoperative vomiting [4].

Both drugs are individually available in market and used to treat nausea vomiting/motion sickness. However, it was found that the combination of CRZ and DPN is more effective than individual ones because both of them have synergistic effects on the other [4].

Numerous analytical techniques were developed to determine both drugs either alone or combined with other drugs, such as liquid chromatography (LC) [5–10], spectrophotometry [11–14], spectrofluorimetry [8,15], capillary electrophoresis [16–18] and electrochemical analysis [19–21], for CRZ and DPN.

By reviewing the literature, it was found that some analytical methods were reported for the determination of both drugs simultaneously, such as spectrophotometry [22,23], spectrofluorimetry [24] and HPLC with UV-detection [25–27]. The proposed method is superior to all reported HPLC methods [25–27] from many different aspects; the first of them is the use of fluorescence detectors, which are not only significantly more selective than UV detectors for many compounds but also more sensitive, stable and easy to use [1]. Furthermore, the development of each of the earlier techniques involved altering a single factor at a time; this made the method laborious, time-wasting, costly, ineffective at fixing errors and liable to produce unpredictable results [28]. However, this study depends on the DoE approach which has emerged as an innovative, systematic strategy that leads to scientifically valid results. The application of this approach in chromatographic conditions optimization has several merits in the efficient development of a rugged and robust technique. Additionally, it is a helpful tool for gathering as much information as possible with fewer experiments. DoE is predicated on looking at several variables at once in order to get a particular response and analysing how they interact [29]. Moreover, method greenness was evaluated and confirmed with four assessment tools. All these merits made the developed method harmonize with the current direction for developing green, simple, sensitive and valid analytical techniques, with less effort, cost, time and solvents for the determination of CRZ and DPN in pure materials, single and combined tablets.

## 2. Experimental

### 2.1. Apparatus and software

— Liquid chromatograph (LC−20 AD) with an injection valve (20 μl sample loop) and a fluorescence detector model RF−10A XL (Shimadzu, Japan) were used for chromatographic determinations.
— Degasser unit (DGU−20A5) was used for the mobile phase degassing.
— Consort pH meter (Turnhout, Belgium) was used for pH adjustment.
— Minitab Software (USA) was used to perform the steps of DoE.

### 2.2. Materials and solvents

— CRZ (100.01 ± 1.10%) and DPN (99.97 ± 1.5%) pure powders, purity as calculated by comparison method [25], were kindly supplied from Repharmed Chemicals Ltd and Pharaonia Pharmaceuticals Industry (Cairo, Egypt).
— Cinnarzine^®^ tablets (25 mg CRZ/tablet) and Dompidone^®^ tablets (10 mg DPN/tablet) are products of Arab Drug Company for Pharmaceuticals and Chemical industries (Al-Amyria, Cairo, Egypt).
— Vertigil^®^ tablets (20 mg CRZ and 15 mg DPN/tablet) were purchased from Cipla Ltd, India.
— Methanol, ethanol and acetonitrile were HPLC grade, and they were obtained from Fisher Scientific, Loughborough, Leics, UK.
— Potassium dihydrogen phosphate was obtained from ADWIC Co. (Cairo, Egypt).

### 2.3. Standard and working solutions

CRZ and DPN stock solutions (200 µg ml^−1^ of each) were prepared individually by dissolving 20 mg of each drug in methanol using a 100 ml volumetric flask. These stock solutions were stable for two weeks when wrapped with aluminium foil and kept in the refrigerator. After that, the mobile phase was used for further dilution to prepare working solutions within the concentration ranges (0.4–5.0 and 0.2–4.5 µg ml^−1^ for CRZ and DPN, respectively).

## 3. General procedures

### 3.1. Construction of the calibration curves

Working solutions were prepared individually by taking different volumes from each drug standard solution into a set of 10.0 ml volumetric flasks and diluting them with the mobile phase. Each solution was injected in a triplicate manner under the optimized chromatographic conditions. Subsequently, each drug peak area was plotted against its concentration to construct a calibration curve and the regression equation was then obtained.

### 3.2. Assay of CRZ and DPN in their synthetic mixtures

Suitable aliquots of CRZ and DPN stock solutions were mixed and diluted with methanol in a 50.0 ml volumetric flask to get a standard laboratory synthetic mixture solution, maintaining the pharmaceutical ratio of 4 : 3 for CRZ and DPN, respectively. Various volumes of this solution were transferred into a series of 10.0 ml volumetric flasks and diluted to volume with the mobile phase. Then, the procedure outlined under §3.1. was followed. By referring to the matching regression equations or using the corresponding calibration curves, the concentrations of each drug were determined.

### 3.3. Analysis of CRZ and DPN in their single commercial dosage forms

Ten Cinnarzine^®^ or Dompidone^®^ tablets were weighed, finely pulverized and mixed well. The accurately measured weight of each drug mixed tablet powder equivalent to 10.0 mg of the respective drug was transferred into a 50.0 ml volumetric flask followed by 20.0 ml methanol. Each flask content was sonicated for approximately 45 min, diluted to the mark with the methanol, then filtered. Furthermore, aliquots containing suitable concentrations within the working range of each drug were then analysed as described in §3.1. Subsequently, each drug concentration was calculated either from a correlative regression equation or the corresponding calibration curve.

### 3.4. Analysis of CRZ/DPN in their co-formulated tablets

Ten Vertigil^®^ tablets were weighed, finely triturated and mixed well. An accurately calculated weight from mixed tablets powder, equivalent to 20 mg of CRZ and 15 mg of DPN, was quantitively transferred to a 100 ml volumetric flask, 70 ml methanol was then added, and flask content was sonicated for approximately 45 min, completed with methanol to the volume and filtered. Furthermore, different volumes from this combined tablet stock solution were accurately transferred into a set of 10 ml volumetric flasks, completed to the volume with mobile phase, then the procedure in §3.1 was implemented with subsequent determination of each drug concentration via the corresponding calibration curves or regression equations.

## 4. Results and discussion

### 4.1. Chromatographic method optimization

Recently, DoE has been adopted to decrease the trial number during the optimization stage and enhance separation quality [29]. Before employing a factorial design, preliminary investigations should be carried out as follows.

#### 4.1.1. Selection of suitable column

Four columns were examined during the experiment as follows:

— Promosil C18 (250 × 4.6 mm, 5 µm).
— Hyper Clone^TM^ C18 (150 × 4.6 mm, 5 µm).
— Hyper Clone^TM^ C8 (150 × 4.6 mm, 5 µm).
— Hypersil^TM^ Phenyl (250 × 4.6 mm, 5 µm).

The first tried column was the most suitable one in terms of time of analysis, peak shape and resolution.

By using other columns, overlapped, broad, blunted or forked peaks were obtained, which is not desirable.

#### 4.1.2. Choice of suitable wavelength

**Figure 2.**
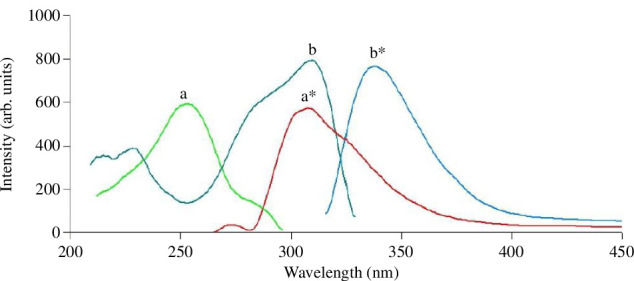
Excitation and emission fluorescence spectra of (a, a^*^) CRZ (4.0 µg/ml) and (b, b^*^) DPN (4.0 µg/ml).

Ideal excitation and emission wavelengths were chosen by analysing the individual fluorescence spectra of CRZ and DPN. It was observed that CRZ exhibits intrinsic fluorescence at λ_ex_ 253.0 nm/λ_em_ 308.0 nm, while DPN has intrinsic fluorescence at λ_ex_ 307.0 nm/λ_em_ 338.0 nm (figure 2). Subsequently, the ideal combination for the excitation and emission wavelengths was 283.0 and 324.0 nm, respectively, since it offered a good compromise for both medication sensitivity.

#### 4.1.3. Mobile phase optimization

In order to investigate the possibility of enhancing the performance of the chromatographic system, a number of changes to the composition of the mobile phase were made. The type and ratio of the organic modifier, pH and ionic strength of the buffer, and flow rate were among these adjustments.

##### 4.1.3.1. pH

The pH of buffer affects the sensitivity, resolution, tailing of the peaks and retention times of the studied drugs. pH range of 3.0–3.8 was carefully selected for factorial design. During the early trials, it was concluded that decreasing pH below 3.0 led to decreasing in the sensitivity and resolution of both drugs and increasing in tailing factors of both peaks. Whereas, by increasing the pH above 3.8, the sensitivity of both drugs also decreased and the retention time of CRZ increased greatly, which is not desirable during this study.

##### 4.1.3.2. The organic solvent type and ratio

During early studies in the screening stage, various solvents like methanol, ethanol and acetonitrile were tried. It was noticed that using ethanol led to overlapped peaks, while using acetonitrile led to an undesired increase in retention times, so methanol was chosen as the suitable organic solvent because its use resulted in the ideal retention times, sensitivity, resolution and shape of peaks. Moreover, its ideal ratio (that was selected for the factorial design study) ranged from 75% to 85%, as decreasing its ratio to less than 75% or increasing more than 85% resulted in long retention times and poor resolution, respectively.

##### 4.1.3.3. Buffer ionic strength

Various ionic strengths of potassium dihydrogen phosphate buffer in the range 0.02−0.06 M were tested. It was concluded that the decrease or increase in ionic strength of the used buffer did not significantly affect retention times and resolution of the two studied drugs, but by increasing the ionic strength to more than 0.02 M, CRZ suffered from forked peaks. Therefore, 0.02 M buffer was selected to be used during this study to get a higher theoretical plate number and good peak resolution. Furthermore, this aqueous buffer has some merits, like simple and easy preparation, and the washing of the column after analysis does not consume a long time. On the other hand, using water led to unresolved and forked peaks.

##### 4.1.3.4. Operating flow rate

The range of flow rate from 0.8 to 1.2 ml min^−1^ guaranteed the ideal separation in a reasonable time. Therefore, this range was selected for the factorial design.

### 4.2. The 2^3^ full factorial design approach

It is a type of design of experiments which is a multivariate optimization. It has a statistics-based approach and offers a number of benefits for developing and optimizing robust methods. The attractiveness of DoE generates from the fact that DoE requires significantly less money, time and resources for optimization than univariate procedures [2]. From early studies, it was found that there were three independent factors affected the chromatographic method performance: pH, percentage of methanol and flow rate; and the responses that were observed were the resolution and drug peak tailing. Therefore, a 2^3^ full factorial design approach (two levels and three independent factors) was adopted during the optimization stage of chromatographic conditions. Eight experiments were suggested by the 2^3^ full factorial design in order to pinpoint the ideal circumstances that produced the optimum response values (table 1). These studied responses were peak resolution between CRZ and DPN (R_s_) and tailing factors of DPN and CRZ peaks.

**Table 1.** Suggested eight runs to perform 2^3^ experimental factorial designs.

run number	% of organic solvent	pH	flow rate
1	75	3.0	0.8
2	75	3.0	1.2
3	75	3.8	0.8
4	75	3.8	1.2
5	85	3.0	0.8
6	85	3.0	1.2
7	85	3.8	0.8
8	85	3.8	1.2

For the determination of three responses (lower, upper and target values), the Minitab response optimizer was used as shown in table 2. Consequently, optimization of the ideal conditions for the input and desirability values was performed [2,30]. Additionally, to make sure that the responses are within acceptable limits, the response optimizer calculates composite desirability (D). Its value ranges from zero to one and it is used to determine whether ideal circumstances are met or not. Maximizing the composite desirability guarantees the establishment of ideal conditions. The Minitab optimization plot displays how each factor affects the composite desirability or responses as well as how those factors interact together to produce the ideal circumstances [2]. Moreover, table 2 explains that in response optimizer, lower, target and upper values are defined for dependent responses. The optimal setting for the input variables along with desirability values is calculated by the Minitab response optimizer. To ensure that the optimum conditions are obtained, the Minitab response optimizer calculates the composite desirability (D), which evaluates whether the responses are in their acceptable limits and range from zero to one. Zero is not accepted as it means that many of the responses are out of their accepted limits, while one means that the condition reached is optimum, so its value is better to be one or near one.

**Table 2.** 2^3^ full factorial design response optimization for reversed-phase high-performance liquid chromatography (RP-HPLC) separation of cinnarizine and domperidone mixture.

the studied parameters							ideal conditions: pH = **3.0** % methanol = **85%** flow rate = 0.8 **ml min^−1^**	
							composite desirability (D) = **0.9486**	
responses	goal	lower	target	upper	weight	importance	predicted responses	individual desirability (d)
resolution	maximize	2.5	2.6	13.28	1	1	2.60	1
DPN peak tailing	minimize	1.31	1.31	2.70			1.33	0.9838
CRZ peak tailing	minimize	1.56	1.56	3.10			1.76	0.8677

#### 4.2.1. Factors that affect resolution (*R*_s_) between CRZ and DPN

**Figure 3.**
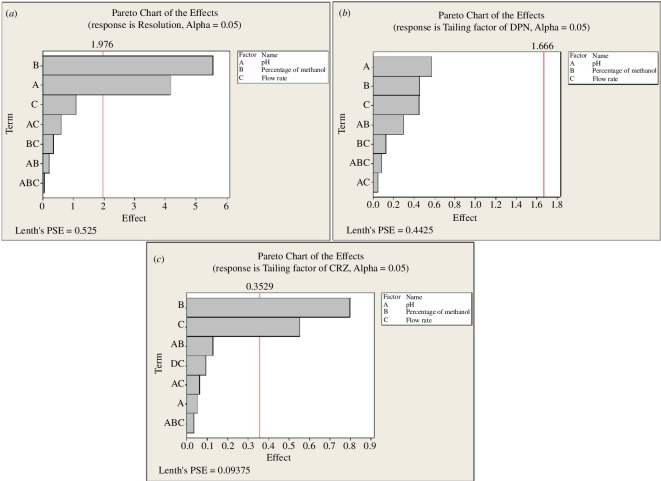
2^3^ Full factorial design Pareto charts of the effects on the chromatographic responses at alpha = 0.05 (*a*–*c*).

**Figure 4.**
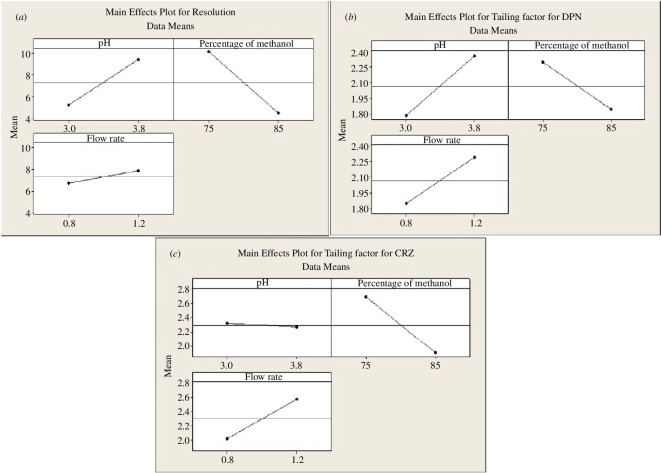
2^3^ Full factorial design main effect plots for chromatographic responses by data means type (*a*–*c*).

**Figure 5.**
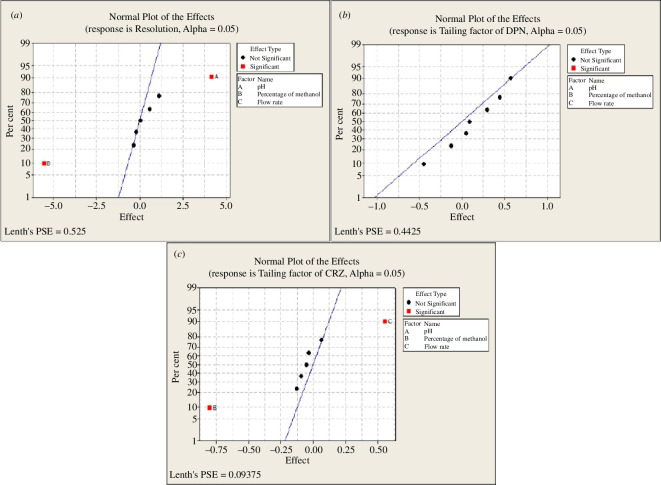
2^3^ Full factorial design normal plots of the effects on the chromatographic responses at alpha = 0.05 (*a*–*c*).

**Figure 6.**
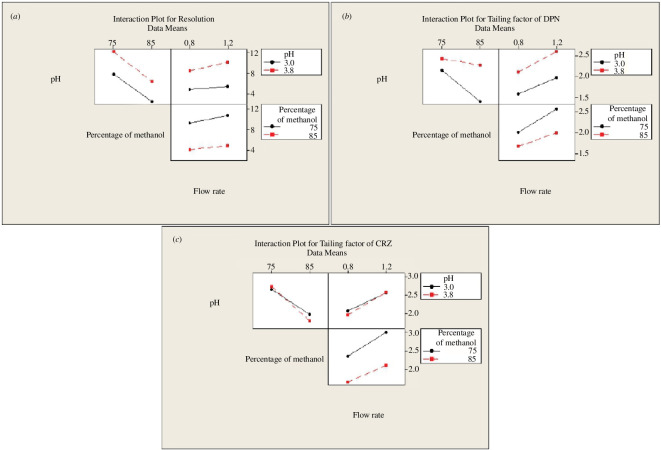
2^3^ Full factorial design interaction plots for chromatographic responses by data means type (*a*–*c*).

According to the Pareto chart of the factor effects (figure 3*a*), the main effects plot (figure 4*a*) and the normal plot (figure 5*a*), the elution buffer pH (A) had a significant positive effect, % methanol (B) had the dominant significant negative influence for a 95% confidence level. From the interaction plots (figure 6*a*), % methanol had a negative relationship with resolution when it interacted with the pH of elution buffer either at low or high levels. Interactions of other included parameters did not significantly affect resolution.

#### 4.2.2. Factors that affect peak tailing of DPN

Pareto chart (figure 3*b*) and the normal plot (figure 5*b*) showed that pH had the highest positive effect on DPN peak tailing, but it was not significant at a 95% confidence level, then % methanol and flow rate. From the main effects plot (figure 4*b*), pH and flow rate had a strong positive impact, while % methanol had a strong negative impact. Moreover, interaction plots represented that pH had a minimum effect at the high level and strongest negative impact at the low level when interacted with % of methanol, and the flow rate had the strongest positive impact at both levels either interacted with pH or % methanol (figure 6*b*).

#### 4.2.3. Factors that affect peak tailing of CRZ

As represented by the Pareto charts (figure 3*c*), the main effects plots (figure 4*c*) and the normal plot (figure 5*c*), % methanol had the strongest significant negative influence on CRZ peak tailing at a 95% confidence level. While flow rate affected it positively. The interaction plot (figure 6*c*) illustrated that % methanol had an inverse correlation on CRZ peak tailing when interacted with the pH at both levels. Furthermore, flow rate had a direct relationship with CRZ peak tailing when interacted with pH or % methanol at both levels for each factor.

### 4.3. Method optimum conditions

**Figure 7.**
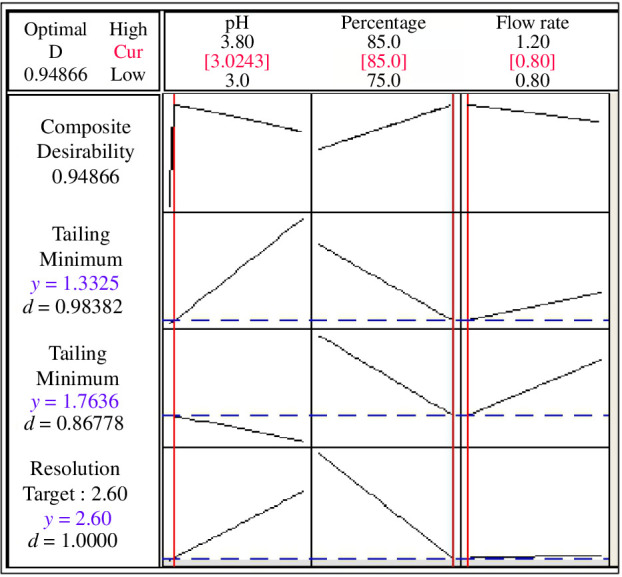
2^3^ Full factorial design optimization plot for the chromatographic parameters.

Consequently, DoE study concluded that the optimum separation conditions were achieved using a mobile phase consisting of methanol and 0.02 M potassium dihydrogen phosphate buffer (85 : 15, v/v, pH 3.0) and 0.8 ml min^−1^ flow rate (figure 7; table 3).

**Table 3.** Optimum chromatographic conditions.

column	Promosil C18 (250×4.6 mm, 5 µm)
mobile phase	methanol and 0.02 M potassium dihydrogen phosphate buffer (85 : 15, v/v)
pH	3.0
flow rate	0.8 ml min^−1^
detector	fluorescence detection at λ_ex_ 283 nm/λ_em_ 324 nm

### 4.4. Method validation

The suggested technique was validated following ICH Guidelines [31] to guarantee method suitability for its desired purpose. The investigated parameters of validation process were as follows.

#### 4.4.1. Linearity and range

Under the optimized experimental conditions, calibration curves for CRZ and DPN were constructed by plotting peak areas versus concentrations of each drug (µg ml^−1^) and it was concluded that the proposed approach was linear within the concentration ranges of 0.4–5.0 and 0.2–4.5 µg ml^−1^ for CRZ and DPN, respectively. Furthermore, linear regression analysis of the data was summarized in table 4 and the following equations were derived:


Peak area = − 427977.64 + 1421987.06 C for CRZ,

**Table 4.** Performance data for the proposed HPLC method for estimation of cinnarizine and domperidone.

parameter	CRZ	DPN
linear working range (µg ml^−1^)	0.4–5.0	0.2–4.5
intercept (a)	−4.2798 × 10^5^	−2.0923 × 10^5^
slope (b)	14.2198 × 10^5^	5.6190 × 10^6^
correlation coefficient (r)	0.9999	0.9999
*N*	6	6
standard deviation of residuals (*S*_y/x_)	3.8298 × 10^4^	9.0310 × 10^4^
standard deviation of intercept (*S*_a_)	2.9369 × 10^4^	7.3061 × 10^4^
standard deviation of slope (*S*_b_)	9.686 × 10^3^	2.5314 × 10^4^
% relative standard deviation (%RSD)	1.064	0.907
% error	0.436	0.369
LOD (µg ml^−1^)	0.068	0.042
LOQ (µg ml^−1^)	0.206	0.130
retention time (minute)	4.5	3.5


Peak area = − 209239.43 + 5619050.20 C for DPN,

where C is the concentration of the studied drug (µg ml^−1^).

#### 4.4.2. Accuracy

The accuracy of the proposed method was confirmed through its application in the quantitative estimation of CRZ and DPN in pure materials and comparing the results with those obtained by comparison method [25] as shown in (table 5). The comparison method recommended HPLC for the simultaneous determination of both drugs using the C18 column. The mobile phase consisted of methanol : 0.05 M phosphate buffer in the ratio (80 : 20 v/v, pH 3.8 adjusted with orthophosphoric acid), flow rate of 1 ml min^−1^ and UV detection at 275.0 nm. Using Student’s *t*‐test and variance ratio *F*-test [32], the data were statistically analysed, and the results revealed no discernible difference between the two methods, verifying the suggested method’s accuracy.

**Table 5.** Analytical data for the analysis of domperidone and cinnarizine in their pure forms using the developed HPLC method.

studied drugs	conc. taken (µg ml^−1^)	% found^a^	comparison method [25] % found^a^
CRZ	0.4	100.75	100.07
	1.0	102.00	100.84
	2.0	100.15	98.44
	3.0	99.40	100.69
	4.0	99.03	
	5.0	100.72	
mean ± s.d.	100.34 ± 1.06		100.01 ± 1.10
*t*‐test	0.47^b^		
*F*-test	1.05^b^		
DPN	0.20	98.00	98.33
	1.25	99.84	102.03
	2.25	100.04	100.08
	3.0	100.00	99.45
	3.75	100.67	
	4.50	99.53	
mean ± s.d.	99.68 ± 0.90		99.97 ± 1.5
*t*‐test	0.38^b^		
*F*-test	2.94^b^		

^a^ Average of three separate estimations.

^b^ The tabulated *t* and *F* values at *p* = 0.05 were 2.30 and 5.40, respectively [32].

#### 4.4.3. Precision

Three separate analyses of each analyte at three different concentrations were performed on the same day in order to assess the intraday precision. To evaluate the interday precision, the same concentrations were measured three times. Small values for s.d. and % relative standard deviation (RSD), as shown in table 6, pointed out that the evolved method has higher precision.

**Table 6.** Precision data of the evolved HPLC method for cinnarizine and domperidone.

drug	conc. (µg ml^−1^)	intraday precision			interday precision		
		mean ± **s.d.**	% RSD	% error	mean ± **s.d.**	% RSD	% error
CRZ	2.0	100.25 ± 0.88	0.88	0.51	99.89 ± 1.13	1.13	0.65
	3.0	99.31 ± 0.94	0.95	0.55	100.48 ± 1.10	1.10	0.63
	5.0	99.58 ± 0.62	0.62	0.36	100.51 ± 0.98	0.98	0.56
DPN	1.50	99.07 ± 1.01	1.02	0.59	99.12 ± 1.05	1.06	0.61
	2.25	99.95 ± 0.36	0.36	0.21	100.35 ± 0.78	0.79	0.45
	3.0	100.01 ± 0.40	0.40	0.23	99.87 ± 0.59	0.59	0.34

#### 4.4.4. Specificity

The specificity of the method was ascertained by evaluating the mentioned analytes in both their commercial single and combined formulations and looking for dosage excipient interferences as shown in tables 7 and 8. It turned out that the assay performance was unaffected by these excipients, and there was no discernible difference between the findings of the estimation of CRZ and DPN in pharmaceutical formulations and pure materials.

**Table 7.** Application of the proposed HPLC method for analysis of cinnarizine and domperidone in their single dosage forms.

parameter	conc. taken (µg ml^−1^)	% found^a^	comparison method [25] % found^a^
Cinnarzine^®^ (25 mg CRZ/ tablet)	2.0	100.13	98.29
	3.0	98.06	101.74
	4.0	99.28	100.09
	5.0	100.96	101.37
mean ± .s.d.	99.61 ± 1.24		100.37 ± 1.56
*t*‐test	0.77^b^		
*F*-test	1.57^b^		
Dompidone^®^ (10 mg DPN/ tablet)	1.5	99.67	99.88
	2.25	100.43	98.21
	3.00	101.36	99.81
	3.75	98.33	101.63
mean ± s.d.	99.95 ± 1.28		99.88 ± 1.39
*t*‐test	0.06^b^		
*F*-test	1.19^b^		

^a^ Average of three separate determinations.

^b^ The tabulated *t* and *F* values at (*p* = 0.05) were 2.44 and 9.27, respectively [32].

**Table 8.** Application of the suggested HPLC method for analysis of cinnarizine and domperidone in their combined dosage forms.

parameters	suggested method				comparison method [25]	
	conc. taken (µg ml^−1^)		% found^a^		% found^a^	
	CRZ	DPN	CRZ	DPN	CRZ	DPN
Vertigil^®^ tablets (20 mg CRZ and 15 mg DPN/tablet)	2.0	1.5	97.94	99.19	98.37	98.57
	3.0	2.25	101.30	98.61	101.10	100.71
	4.0	3.0	100.34	101.00	99.63	101.42
	5.0	3.75	99.39	100.68	100.12	99.64
mean			99.74	99.71	99.90	99.34
s.d.			1.43	1.32	1.59	1.53
*t*‐test			0.06^b^	0.54^b^		
*F*-test			1.59^b^	1.76^b^		

^a^ Average of three separate determinations.

^b^ The tabulated *t* and *F* values at (*p* = 0.05) were 2.44 and 9.27, respectively [32].

#### 4.4.5. Robustness

The process parameters, such as the pH (3.0 ± 0.1), percentage of methanol (85% ± 1) and the used buffer ionic strength (0.02 M ± 0.01), were deliberately altered to evaluate the robustness. The findings indicate that the HPLC approach is robust as these variations had little to no effect on its performance (table 9).

**Table 9.** The proposed method robustness.

parameter	mean ± s.d.		% RSD		retention time	
	CRZ	DPN	CRZ	DPN	CRZ	DPN
pH (3.0 ± 0.1)	99.48 ± 1.28	99.28 ± 1.14	1.29	1.15	4.5 ± 0.2	3.5 ± 0.04
methanol ratio (85% ± 1)	99.89 ± 1.13	100.21 ± 0.83	1.13	0.83	4.5 ± 0.09	3.5 ± 0.03
buffer strength (0.02 M ± 0.01)	100.48 ± 1.10	99.41 ± 0.54	1.10	0.55	4.5 ± 0.01	3.5 ± 0.001

#### 4.4.6. System suitability

System suitability assessments were done referring to ICH Guidelines [31] on mixture of CRZ and DPN to calculate the chromatographic parameters, as represented in table 10.

**Table 10.** System suitability parameters of the proposed HPLC method.

parameter	DPN		CRZ
number of theoretical plates (NTP)	990		1773
capacity factor (k`)	0.67		1.14
resolution (R_s_)		2.6	
tailing factor (T)	1.3		1.7

### 4.5. Comparison of analytical performance of the suggested and previously reported methods

The suggested method was compared with the previously reported methods regarding LOD, LOQ, range, experimental conditions, application and greenness assessment as represented in table 11.

**Table 11.** Analytical performance of the suggested technique and other reported methods.

method	range (µg ml^−1^)		LOD (µg ml^−1^)		LOQ (µg ml^−1^)		experimental conditions	application	greeness assessment
	CRZ	DPN	CRZ	DPN	CRZ	DPN			
this method	0.4–5.0	0.2–4.5	0.068	0.042	0.206	0.130	as mentioned in §4.3.	single and combined dosage forms	four assessment methods
[22]	5.0–30.0	5.0–30.0	0.039	0.093	0.128	0.308	direct measurement of drug absorbance using methanol as solvent and 1% phosphoric acid as diluting solvent	combined dosage forms	no assessment
[23]	0.5–34.0	0.5–36.0	0.266	0.227	0.885	0.755	ion-pair complex formation with rose Bengal dye in buffer solution (pH 5.0).	single dosage forms	no assessment
[24]	0.1–1.3	0.1–3.0	0.017	5.77 × 10^−3^	0.058	0.020	second derivative synchronous fluorometric measurement using aqueous methanol (50% v/v)	synthetic mixtures and combined dosage forms	no assessment
[25]	10.0–35.0	5.0–30.0	0.067	0.221	0.051	0.169	HPLC method using C18 column, mobile phase consisted of methanol: 0.05 M phosphate buffer (80 : 20 v/v, pH 3.8) and UV detection	combined dosage forms	no assessment
[26]	24.0–56.0	18.0–42.0	—	—	—	—	HPLC method using C18 column, mobile phase consisted of phosphate buffer : acetonitrile : methanol (40 : 40 : 20, pH 3.0) and UV detection	combined dosage forms	no assessment
[27]	0–36.0	0–5.5	0.615	0.462	1.23	0.925	HPLC method using C18 column, mobile phase consisted of orthophosphoric acid : acetonitrile (70 : 30) and photo diode array (PDA) detection	combined dosage forms	no assessment

## 5. Method applications

### 5.1. Analysis of CRZ/DPN in their synthetic mixtures

**Figure 8.**
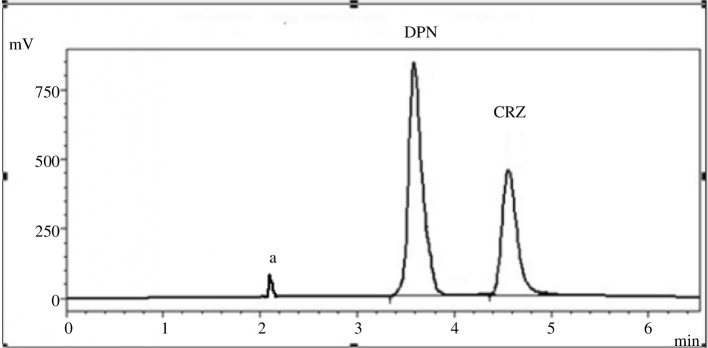
Typical chromatogram of a synthetic mixture of CRZ (4.0 µg ml^−1^) and DPN (3.0 µg/ ml^−1^) under the studied chromatographic conditions where (a) is the solvent front.

The procedure was effectively applied for the analysis of CRZ and DPN in synthetic mixtures (figure 8). A statistical evaluation of the acquired data using the variance ratio *F*-test, and Student’s *t*‐test [32] revealed no discernible distinction in the accuracy and precision of the two approaches’ performance as displayed in table 12.

**Table 12.** Application of the evolved HPLC method for analysis of cinnarizine and domperidone in their synthetic mixtures.

parameters	suggested method				comparison method [25]	
	conc. taken (µg ml^−1^)		% found^a^		% found^a^	
	CRZ	DPN	CRZ	DPN	CRZ	DPN
	2.0	1.5	99.78	98.60	99.00	98.01
	3.0	2.25	98.06	99.40	101.89	100.88
	4.0	3.0	99.81	99.22	100.59	101.75
	5.0	3.75	101.95	101.63	98.73	99.92
mean			99.80	99.87	100.05	100.14
±s.d.			1.13	1.15	1.47	1.60
*t*			0.14^b^	0.41^b^		
*F*			1.17^b^	1.47^b^		

^a^ Average of three separate estimations.

^b^ The tabulated *t* and *F* values at (*p* = 0.05) were 2.44 and 9.27, respectively [32].

### 5.2. Analysis of CRZ/DPN in their single commercial dosage form

The procedure was successfully used to determine DPN and CRZ in single dosage forms. As demonstrated by *t* and *F* values [32], there was no significant distinction between the obtained data and those attained from the comparison method [25] as listed in table 7. Subsequently, there was no evidence of excipient interference.

### 5.3. Analysis of CRZ/DPN in their co-formulated tablets

**Figure 9.**
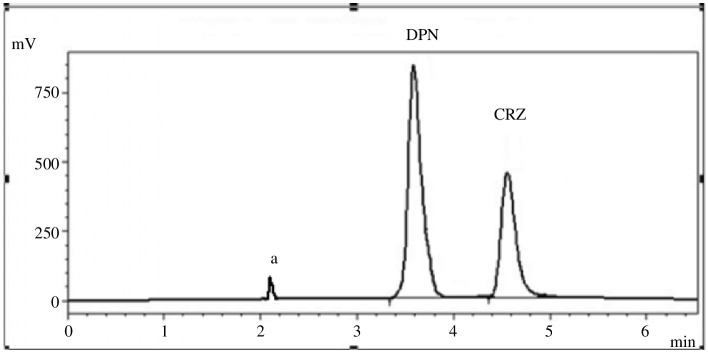
Typical chromatogram of co-formulated tablet of CRZ (4.0 µg ml^−1^) and DPN (3.0 µg ml^−1^) under the studied chromatographic conditions, where (a) is the solvent front.

The investigated medications were analysed in their combined tablets (figure 9), and table 8 shows that the assay produced satisfactory results which agreed well with that of the comparison method [25].

## 6. Greenness assessment

Green analytical chemistry has made progress in quantifying how analytical techniques affect the environment. The assessment of the greenness of chemical processes is one of the challenges encountered in green chemistry. It is widely recognized that processes that are unmeasurable are uncontrollable. So, four green chemistry metrics such as green analytical procedure index (GAPI), analytical greenness (AGREE), national environmental methods index (NEMI) and analytical eco-scale score were applied to assuring eco-friendliness of the evolved method.

Recently, GAPI has evaluated the green character of an entire analytical methodology. It is applied using pentagrams, which employ a three-level colour system with green, yellow and red denoting low, medium and high environmental effects [33], respectively, as shown in table 13.

**Table 13.** Evaluation results of the greenness of the suggested method.

(1) green analytical procedure index (GAPI)		
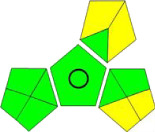		
(2) NEMI pictogram	(3) AGREE pictogram	
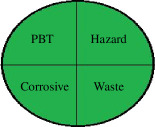	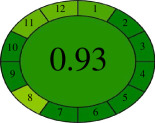	
(4) analytical eco-scale score		
item		penalty points
1-reagent		
methanol		6
0.02 M phosphate buffer		0
2-HPLC-fluorimetric detection		2
3-occupational hazard		0
4-waste		3
total penalty points		11
analytical eco-scale score		89

Moreover, NEMI is characterized by its simplicity and readability for potential process users. It only takes a quick look at the NEMI pictogram to get a general overview of how a technique will affect the environment [34] as presented in table 13.

The AGREE graph has 12 sectors encircling its circumference and is shaped like a clock. Each sector represents one of the 12 principles of green analytical chemistry (GAC). The method performance with respect to each of the GAC principles is shown by a red, orange and green scale [35]. The total performance of the method is represented by the colour in the centre of the graph, which has a score between 0 and 1. The score of this suggested method was 0.93 as shown in table 13.

The analytical eco-scale is another tool for the assessment of analytical methods’ ecological impact [34]. For each method, parameter penalty points are assigned. The sum of penalty points should be subtracted from the basis of 100. The closer the score to 100, the higher degree the greenness obtained (table 13).

## 7. Conclusion

It was crucial to develop a straightforward, accurate, selective, robust and precise HPLC technique for the simultaneous assay of CRZ and DPN in pure, single and co-formulated pharmaceutical forms. This is the first chromatographic technique using a fluorescence detector to analyse both medications simultaneously in approximately a short time of analysis, less than 6 min. A two-level full factorial design was used to optimize the chromatographic conditions, which cut down on time and trial count. Moreover, greenness, sensitivity, rapidity, appropriate robustness and wide applicability distinguished the approach and made it successfully implemented in actual-life situations by analysing both medications in their single and combined commercial dosage forms, with satisfactory percentages found without any interference. Ultimately, this method can be followed effectively in quality control work.

## Data Availability

The datasets generated during and/or analysed during the current study are available in the Dryad repository [36].
